# A myth in language teacher learning: Lesson observation

**DOI:** 10.3389/fpsyg.2022.1026833

**Published:** 2022-10-20

**Authors:** Min Gu

**Affiliations:** School of Foreign Languages, Zhejiang Sci-Tech University, Hangzhou, China

**Keywords:** teacher learning, lesson observations, cognitive processes, observation foci, Chinese senior high school EFL teachers

## Abstract

This study explores the learning process of 32 Chinese senior high school English as a foreign language (EFL) teachers *via* three demonstration lessons. It was demonstrated *via* a data analysis of oral reports and interviews that the cognitive activity of “question,” which was considered a significant contributor to collaborative discussion, was seldom involved in the participating teachers’ learning process, and that the absence of this cognitive activity reduced their learning to individual study of the observed practical skills. The study further reveals four factors that prevented the participating teachers from collaboratively constructing language teaching knowledge based on what was observed: These were (1) their perceived purpose of the modeled lessons, (2) their manner of making meaning, (3) their understanding of observer–observed relationship, and (4) their perception of professional learning. The analysis presented provides important insights for teacher educators to better facilitate in-service teachers of foreign languages learning through observation.

## Introduction

Despite the long-standing role of lesson observation as a crucial means of teacher education in language teacher educational programs (Day, 1990; Vélez-Rendón, 2002; Richards and Farrell, 2005; Xu, 2017), little is known with regard to how language teachers actually learn via observation. In particular, the research on language teachers’ focus of observation and their processing of the observed information is sparse. To help bridge this gap, the present study examines the learning processes of 32 Chinese senior high school English as a foreign language (EFL) teachers via three demonstration lessons. By investigating their way of directing attention, processing information, and making sense of what they have observed, this study attempts to uncover knowledge about the nature of in-service EFL teachers’ learning through observation. Two research questions guide this inquiry:

1. In what manner did the participating teachers learn from the three demonstration lessons?
2. What factors had an effect on their learning through observation?

## Lesson observation

Lesson observation is a key constituent of teacher education programs (De Paor, 2015). Whether it is in the context of an in-service training program, an initial teacher training course, or a collaborative professional development initiative, observation has been commonly used as an important tool for developing teachers’ skills and knowledge (O’Leary, 2014).

In language teaching, as Richards and Farrell (2005) argue, observation can lead to both enhanced awareness of what other teachers do in the classroom and how they do it, and discovery of effective teaching strategies as well as new ideas for solving problems in teaching. Existing research has highlighted the fact that modeled lessons hold the potential to foster language teachers’ professional learning (e.g., Grierson and Gallagher, 2009; Chien, 2015; Yuan, 2018). Grierson and Gallagher (2009), in their qualitative study on the experiences of literacy teachers in a demonstration classroom professional development initiative, concluded that demonstration classrooms can act as a catalyst for change in the observing teachers’ beliefs and practices. In terms of pre-service teachers, the study of Chien (2015) revealed that the observation of expert teachers’ instructional practices helped pre-service English teachers develop their pedagogical content, pedagogical content knowledge, and classroom management skills. More recently, Yuan (2018) argued—based on an investigation of an experienced EFL teacher educator’s modeling practice—that the use of modeling played a pivotal role in linking theories with practice, in turn promoting students’ learning to teach.

In addition to a tool for learning skills and knowledge, there has been growing interest in the value of lesson observation as a means to stimulate critical reflection (Brookfield, 1995) and reflective dialog (O’Leary and Price, 2017). Armitage et al. (2003) make the point that when observation is used as part of a teacher education program, it can be “the basis of some of the most useful professional reflection you can undertake in order to improve performance” (p. 47). For such teacher development to take place, O’Leary (2014) argues that certain conditions need to be established. One of the key factors is a teacher’s willingness to adopt an inquiry-based stance to learning through observation, which typically involves teachers questioning what they observe and challenging assumptions by learning about and experimenting with different approaches to teaching. As Dymoke and Harrison (2008) note, “it is through both questioning and investigating that reflection has the potential to lead to a developing understanding of professional practice” (p. 8). Another essential factor that O’Leary refers to is a democratic and egalitarian observer–observed relationship, which is considered fundamental in facilitating learning through observation. Meanwhile, he also contends that in order for teachers to engage with observation as a tool for reflective practice, they must be encouraged to co-construct knowledge through post-observation “collaborative discussion where thoughts and ideas about classroom practice are first articulated and then reformulated in a progression towards enhanced understanding” (Walsh, 2016, p. 121).

In recent years, a number of studies in TESOL teacher education have focused on how to help teachers engage in reflective practice after observation. Some studies have demonstrated that mentors/trainers tend to employ specific interactional techniques to encourage reflective dialogue, for instance, *via* the use of scaffolding (Engin, 2015), questioning techniques (Le and Vásquez, 2011), as well as mentor comments, assessments, and advice to trigger teacher reflection (Waring, 2013). Others have disclosed that reflective tools are increasingly used to enhance professional understanding in lesson observation, such as post-lesson discussions with peers (Yuan and Lee, 2014), online chats, and discussion forums used for post-observation discussions (Farr and Riordan, 2012), and video recordings in reflective feedback sessions (Eröz-Tuğa, 2013).

While there are a variety of accounts describing the facilitative role of mentors/trainers in EFL teachers’ post-observation reflection, there appear to be few that detail EFL teachers’ learning through observation, e.g., how EFL teachers direct their own attention, process the information regarding teaching and learning in the modeled lessons, and make sense of the observed classroom practice. Insight into this learning process may not only provide opportunities for teachers to reflect on and transform their own learning processes, but also have strong implications for effective mentorship in observation.

## Theoretical perspectives

The present study draws on model of learning of Moon (1999) as an analytical framework in order to investigate the learning processes of the participating teachers in the observation sessions. To gain further insight into their observation foci, conceptualization of a knowledge base of Freeman (2016) for EFL teachers is also introduced subsequently.

### Stages of learning

The primary tool for the analysis was map of learning of Moon (1999), which divides the process of learning into five stages. The first stage entails the initial sensory encoding of the learning material, and is referred to as *noticing* (Moon, 1999, p. 139). Moon (1999) identifies four factors that impact the way a learner’s attention is directed: (1) previous knowledge and experience, (2) perceived and given purpose of learning, (3) constitutive factors such as emotion and motivation, as well as (4) the amount of attention that learners draw to the material. In the present study, noticing is the stage concerned with what the participating teachers attended to in their observations, which the author further examines using framework of Freeman (2016) for an EFL teacher knowledge base.

The second stage, *making sense*, is a process in which learners seek some coherence in the material of learning (Moon, 1999, p. 142). This type of processing requires little connection between the new material and what learners already know. Consequently, the representation of learning at this stage will mainly demonstrate surface processing with “ideas not well linked” (Moon, 1999, p. 142).

Meaningful learning occurs as a result of the third stage, *making meaning*, which refers to the process of assimilating the material of learning into a learner’s cognitive structure (Moon, 1999, p. 143). Although Dewey (1910) does not directly use the term making meaning, he also represents this process in his work, arguing that perplexity or doubt is a necessary catalyst for the creation of meaning and further developing a distinction between the creation of meaning *via* analogous experience and the creation of meaning by systematic inquiry. According to his view, if learners draw solely on prior knowledge or past experience to immediately settle doubts, they will have only engaged in uncritical thinking, or in words of Moon (1999), surface learning, but if learners engage in systematic inquiry to remove doubts, which means to hunt for new material to prove or refute what is already known, they will develop reflective thinking, or what Moon (1999) terms deep learning. Cochran-Smith and Lytle (2009) emphasize that a systematic and intentional inquiry into practice also entails teachers building knowledge together. The creation of meaning, therefore, can also be viewed as a collaborative act rather than solely an individual one.

Until the stage of making meaning, the original material of learning has been processed and has become part of the cognitive structure itself. In the subsequent stage of *working with meaning*, learners reprocess or reformulate the knowledge resulting in ongoing learning (Moon, 1999, p. 144). In other words, the stage of working with meaning serves as a means to pursue further accommodation of cognitive structure away from the source of the original learning.

The last stage, *transformative learning*, represents “a more extensive accommodation of the cognitive structure” (Moon, 1999, p. 146). In the view of Moon (1999), there appears to be no qualitative difference between the stages of working with meaning and transformative learning.

### EFL teacher knowledge base

Drawing on Ball et al. (2008), Freeman (2016) distinguishes between four types of EFL teacher knowledge: (1) disciplinary knowledge, (2) knowledge of pedagogy, (3) knowledge-in-person/knowledge-in-place, and (4) knowledge-for-teaching. *Disciplinary knowledge* refers to the knowledge that is needed to teach languages, including knowledge about linguistics, psychology, literature, sociology, and anthropology. *Knowledge of pedagogy* deals with how to apply disciplinary knowledge in language teaching. These two kinds of knowledge are generated by outside experts and researchers and can best be learned through “a mixture of transmission and organized practice” (Freeman, 2016, p. 62).

The third type of EFL teacher knowledge consists of two aspects: *knowledge-in-person* and *knowledge-in-place*. The former centers on how teaching knowledge is enacted by a teacher and comes to be known as *personal practical knowledge* (PPK; Clandinin and Connelly, 1986), while the latter focuses on how the situation of a particular classroom shapes a teacher’s decision-making process and is referred to as *pedagogical content knowledge* (PCK; Shulman, 1987). It is assumed in the third type that knowledge belongs to master teachers (Wallace, 1991; Cochran-Smith and Lytle, 2009), who will transmit this knowledge to others *via* teaching demonstrations.

Finally, *knowledge-for-teaching* is concerned with the purpose underlying teacher agency. According to Freeman (2016), student learning is a common purpose that drives teaching practices in different settings. Unlike the first three types, where knowledge is seen as an external entity that “can be defined, explained and passed intact” (Deyrich and Stunnel, 2014, p. 90) from one person to another, this fourth type views knowledge as “a shared asset whose meaning is negotiated and mediated by both parties” (O’Leary, 2014, p. 119). It is this epistemological view with regard to knowledge that opens up the possibility of better understanding through collaborative discussion how teaching creates opportunities for student learning.

## Research methodology

As Johnson (2003) has stated, verbal reporting has the potential to make covert processes overt. Thus, verbalization was deemed valid in this study to elicit the observation foci of the participating teachers and also yield significant information about the cognitive processes involved in their learning. In addition, semi-structured group interviews were mainly used to (1) triangulate the verbal reporting data and (2) explore what factors had an effect on the participating teachers’ learning.

### Research design

Lesson observation in the present study lasted for 3 weeks. Each week, the participating teachers were invited to observe one demonstration lesson, which applied the use of communicative tasks as its main mode of instruction. These lessons were given by an expert-teacher educator, who was also leading a team for developing the local English textbooks that were intended for use in the following year. This expert-teacher educator was quite familiar with the participating teachers because he was frequently involved in teacher development programs in the local educational district. As a team member, the author was invited by the expert-teacher educator to observe the demonstration lessons as well. Following each lesson, the participants were randomly divided into small groups to have discussions for about 15 min, a session that was carried out “in light of the group’s experience and beliefs” (Richards and Farrell, 2005, p. 53) about how to teach reading, writing, and grammar. Subsequently, a verbal reporting session was organized for the representatives from each group to present their thoughts and ideas about the observed classroom practice, which was followed by the educator’s feedback on the concerns expressed by the representatives in their debriefing.

### Participants and settings

The participants were 32 in-service EFL teachers recruited through a teacher-training program, which was part of a curriculum reform initiative organized by the local district, in Shanghai, China. The primary aim of the training program was to demonstrate how to use communicational activities, such as teacher–student interactions for teaching reading comprehension, writing, and grammar, in the three demonstration lessons, respectively.

All of the senior high schools (nine in all) in the district were enthusiastic about this educational activity as they knew that they were going to use the new textbooks in about 2 years’ time. Thirty-two participating teachers from the nine schools (see Table 1) were verbally informed that the purpose of this study was to examine how teachers learn through observation.

**Table 1:** Number of participants from the nine schools.

Level of school	Number of schools	Number of participants
Municipal key	2	8
District key	2	10
Regular	5	14

All of the participants agreed to be audio-recorded in the discussion and receive interviews and visits to their classrooms in the subsequent investigations.

### Data collection

#### Verbal reporting

In seeking to reflect as naturally as possible how the participating teachers learned through observation and equally to reduce disturbances to their mental processes, the verbalization methodology employed in the present study was based on non-mediated verbal reporting of Green (1998), where the participants were free to say whatever came into their minds and there were not any interventions until the end of their debriefing.

All the verbalizations were audio-taped, resulting in 18 audio-recordings from 17 participating teachers (one teacher reported on two observed lessons). The recordings varied in time from 3 to 7 min and totaled about 100 min.

#### Semi-structured group interviews

Semi-structured group interviews were designed for the following reasons: (1) to collect different sources of data to promote “convergent validity” (Cohen et al., 2011), (2) to investigate the factors affecting the participating teachers’ learning through observation, and (3) to gather information about whether learning through observation made a difference to their teaching practices. In light of the last two reasons, the interviews were organized 3 months later after lesson observation with the intension of providing enough time for the author to conduct data analysis so as to gain some insight into the way the participating teachers learned through observation and also for the participating teachers to put into practice what they had learned from the demonstration lessons.

Of the nine schools involved in the training program, three were selected for the interviews. The criteria for selecting these schools were that they represented the three levels of schools (municipal key school, district key school, and regular school) in the district, and the number of participating teachers in the three schools was the largest among those of others the same level. There should have been 17 participants in all for the three school-based group interviews, but two participants from the municipal key school were on sick leave and hence absent from the interview.

The outline of the interview questions (see Appendix) was sent to the participants 3 days before the interviews. Finally, the interview data included three audio-recordings, each lasting about 1 h 30 min.

### Data analysis

Verbal reports and interviews were transcribed verbatim for analysis. The transcripts of the verbal reports were subjected to protocol coding of Saldana (2013), in which the data were coded “according to a pre-established, recommended, standardized, or prescribed system” (p. 151). Later, inductive content analysis (Krippendorff, 2013) was employed to analyze the transcripts of the interviews.

At the very beginning, the author coded and analyzed all the transcripts alone. Once she finished coding the verbal reporting data, the initial codes and the unitized transcripts were shared with a researcher of second language teacher education who was invited to check the codes and the units one by one to ensure that they were analyzed appropriately. Disagreements regarding categorization of units, names, and definitions of codes, as well as the relationship between codes, were discussed thoroughly until an overall agreement was reached between the two researchers. During the coding of the interview data, the author checked her interpretation with the participating teachers to verify the analysis.

#### Analysis of the data from verbal reporting

Three stages of data analysis were performed on the 18 transcripts from verbal reporting. The first stage aimed at setting up a coding system for EFL teacher knowledge in light of classification of language teaching knowledge of Freeman (2016). Table 2 presents the four codes proposed by Freeman (2016).

**Table 2:** Codes with descriptions and illustrative examples from the verbal reporting data.

Code	Description	Illustrative example
Disciplinary knowledge	Knowledge needed to teach languages, including knowledge about linguistics, psychology, literature, sociology, and anthropology	“The teacher drew students’ attention to the difference in pronunciation between British English and American English” (V3T2U7^a^)
Knowledge of pedagogy	Knowledge concerning how to teach languages, including knowledge about teacher disposition, lesson plan, classroom management, and teaching techniques	“The teacher caught students’ attention by posing questions to the whole class rather than to a particular one” (V2T6U5)
Knowledge-in-person/knowledge-in-place	The way that language teaching knowledge is enacted by a teacher in the classroom	“The teacher taught vocabulary of the reading passage in different ways, such as paraphrasing, using body language and setting up context” (V1T5U4)
Knowledge-for-teaching	The purpose that shapes how language teaching knowledge is used	“What’s the purpose of deep reading? In doing it, should teachers place emphasis on the forms that students produce or on the text message to sharpen their understanding? I think it’s hard to achieve both at the same time” (V2T3U2)

a The abbreviations “V” for a verbal reporting session, “T” for a teacher who verbalized thoughts on the observed lesson, and “U” for a unit of analysis are used to identify quotes from the verbal reporting data. For example, the abbreviation “V3T2U7” refers to a quote from the seventh unit of analysis in the verbal report of a teacher who was the second one to articulate thoughts during the third verbal reporting session.

In the second stage, the focus was specifically on identifying units of analysis that represented EFL teacher knowledge in each transcript. Some principles employed by Ellis and Barkhuizen (2012) for deciding on the units of analysis were applied: A unit was identified by looking for a keyword or phrase in relation to the coding scheme in Table 2; the length of a unit was limited to the sentence or sentences in which a particular aspect of EFL teacher knowledge was expressed.

The third stage involved coding the units of analysis from two perspectives: On the one hand, the coding took place following the coding system for EFL teacher knowledge (see Table 2) in an attempt to explore the observation foci of the participating teachers; on the other hand, the units were recoded with a list of codes established for cognitive activities with the purpose of examining what cognitive processes tended to be employed in the process of the participating teachers attending to a specific type of EFL teacher knowledge.

The codes for cognitive activities were developed by modifying operator scheme of Johnson (2003), where operators are described as verbs representing cognitive activities. Table 3 shows the operators in the present study alongside a short description of each. Some of the operators were borrowed from protocol of Johnson (2003) and others emerged from the data.

**Table 3:** Operators and their descriptions.

Operator	Description
Analyze Assume Consider Describe Evaluate Explain Figure out Inspire Propose Question Resort	Subject to an analysis in order to gain a better understanding of teaching and learning Make an assumption (e.g., about the learning process) Draw attention to and reflect on the challenge informed by the observed class Give a descriptive account of what is observed Consciously attempt to reach a judgement on the teaching practice in the observed class Clarify the purpose or doubt about the teaching practice in the observed class based on one’s prior knowledge Try to find solutions to doubts about the teaching practice in the observed class by deep thinking when prior knowledge is not available Get new ideas about foreign language teaching from the observed class Suggest a possible solution to the problem identified in the observed class Express doubts about the purpose behind the teaching practice in the observed class, usually in the form of a question Seek help for the potential problems in the observer’s teaching practices

As mentioned earlier, the cognitive activities were coded based on units of analysis reflecting EFL teacher knowledge, whereby more than one operator would be assigned to a unit of analysis in which a particular type of EFL teacher knowledge was articulated.

Furthermore, based on model of learning of Moon (1999), the operators can be further divided into three categories. For example, the first category contains the operator of “describe,” which enabled the participating teachers to process what they observed into the stage of making sense. The first category, therefore, demonstrates the qualities of surface approaches to processing.

The second category is comprised operators like “explain,” “question,” and “figure out,” which opened up the possibility for the participating teachers to reach the stage of making meaning. Albeit within the same category, the three operators play different roles in the process of making meaning. “Explain” underlines making sense of the new material on the basis of prior knowledge, thus perhaps leading the participating teachers to understand what they observed at face value, while “question” and “figure out” stress the grasping of meaning through further inquiry, hence likely bringing about a deeper understanding of the observed class. Furthermore, the difference between “figure out” and “question” lies in the fact that the former dips back into what is observed for additional evidence, whereas the latter intends to search for more details through dialogue with the observed teacher. As such, “question” is more likely to trigger collaborative discussion.

The remaining operators, i.e., “assume,” “analyse,” “inspire,” “evaluate,” “resort,” “propose,” and “consider,” belong to the third category of working with meaning or transformative learning, which allows the participating teachers to manipulate meaningful knowledge toward a particular purpose. The operators in this category are described by Moon (1999) as tools of manipulation of knowledge.

Since the stages of making sense and making meaning are the basis for that of working with meaning or transformative learning, operators in the first two categories were the focus of investigation in this study.

#### Analysis of the interview data

With the help of inductive content analysis (Krippendorff, 2013), which involves reducing data into manageable representations by means of unitization, coding, comparison, and categorization, four themes emerged in the process of analyzing the three interview recordings: These were (1) focus of observation, (2) reasons for observation foci, (3) impact of learning through observation on teaching practices, and (4) explanation for (not) using a certain cognitive activity. The results from the interview data are used directly in the discussion section to address the second research question and also to ensure an interpretation of the findings.

## Results

In this part, I describe the results from the data analysis of verbal reporting, with primary attention paid to (1) the cognitive activities involved in the participating teachers’ learning processes and (2) the cognitive activities employed for different types of EFL teacher knowledge.

### The results of cognitive activities in general

The operators denoting the cognitive activities are presented in Table 4.

**Table 4:** The participating teachers’ cognitive activities.

Category of processing	Operator	NOO^a^	Quote from the verbal reporting data
Surface processing	Describe Explain	76 19	“The teacher presented the text structure step by step with the help of the blackboard and by the end of the class he underlined the key points on the blackboard with red chalk” (V1T3U6^b^) “In deep reading, the teacher shot questions at students to help them make a detailed inquiry into the scenes selected from the passage” (V2T5U2)
Deep processing	Figure out Question	4 1	“At first, I found the transition from deep reading to mini-project was a bit fast. On second thoughts, I guessed there might be some connections between the two activities, that is, the thinking pattern developed in deep reading could be applied to mini-project” (V2T3U7) “What’s the purpose of deep reading? In doing it, should teachers place emphasis on the forms that students produce or on the text message to sharpen their understanding? I think it’s hard to achieve both at the same time” (V2T3U2)
Manipulation of knowledge	Evaluate Inspire Consider Resort Analyze Propose Assume	35 18 16 11 7 4 2	“So I think the blackboard was used very effectively” (V1T3U6) (when talking about the observed teacher’s classroom questioning) “To ask good questions we should anticipate students’ answers at the time of lesson planning and meanwhile prepare several follow-up questions in case that students’ answers are not satisfying” (V2T2U3) “Giving immediate corrective feedback to students’ writing as the teacher did in his class makes high demands on us” (V3T2U11) “In deep reading, if students fail to give a satisfactory answer to my question, what should I do? Can you give me some suggestion?” (V2T5U7) “PPT is not conducive to classroom interaction because it confines the communication to a list of questions prepared beforehand” (V1T6U5) “Before writing, it would be better for the teacher to present some language support on the PPT” (V3T4U1) “During the presentation, the student who acted as the questioner in the latter group might copy the questions of the former group rather than think for himself” (V2T1U2)

a NOO = number of occurrences.

b The abbreviations “V” for a verbal reporting session, “T” for a teacher who verbalized thoughts on the observed lesson, and “U” for a unit of analysis are used to identify quotes from the verbal reporting data. For example, the abbreviation “V1T3U6” refers to a quote from the sixth unit of analysis in the verbal report of a teacher who was the third one to articulate thoughts during the first verbal reporting session.

The results illustrate that “describe” is the dominant cognitive activity (76 times) with “question” being the least-used (one time). Moreover, the operators for surface processing, i.e., “describe” and “explain” (19 times), occur 95 times in all, while those for deep processing, i.e., “question” and “figure out” (four times), appear merely five times in total. This is interpreted to mean that the participating teachers processed the observed teaching practice on a superficial level most of the time and thus the cognitive activities in the third category (i.e., manipulation of knowledge), which includes “assume,” “analyse,” “inspire,” “evaluate,” “resort,” “propose,” and “consider,” were largely due to surface processing.

### The results of cognitive activities for specific teacher knowledge

Table 5 displays the EFL teacher knowledge that the participating teachers attended to and the cognitive activities that they employed for different types of EFL teacher knowledge.

**Table 5:** Cognitive activities for different types of EFL teacher knowledge.

EFL teacher knowledge	NOUOA^a^	Cognitive activity
Disciplinary knowledge	12	describe (8^b^); **assume**^c^ (2); analyse (2); reflect (1); evaluate (1); inspire (1); resort (1); propose (1)
Knowledge of pedagogy	35	describe (22); evaluate (15); reflect (5); inspire (4); consider (4); analyse (3); resort (2); propose (1)
Knowledge-in-person/Knowledge-in-place	42	describe (24); inspire (10); evaluate (8); resort (8); consider (3); propose (2); reflect (2); analyse (2)
Knowledge-for-teaching	24	describe (22); **explain** (19); evaluate (11); **figure out** (4); inspire (3); consider (1); **question** (1)

a NOUOA = number of units of analysis.

b The number of times a cognitive activity was used for a particular type of EFL teacher knowledge is presented in parentheses.

c The cognitive activities that were employed exclusively for a particular type of EFL teacher knowledge are in bold.

As the table shows, the observation foci of the participating teachers include the four types of EFL teacher knowledge. To be specific, there are 12 units of analysis relevant to disciplinary knowledge, 35 in relation to knowledge of pedagogy, 42 in connection with knowledge-in-person/knowledge-in-place, and 24 related to knowledge-for-teaching. The results indicate that the participating teachers paid primary attention to practical skills related to knowledge of pedagogy and knowledge-in-person/knowledge-in-place.

Another characteristic evident from Table 5 was that some cognitive activities were seemingly employed exclusively for a particular type of EFL teacher knowledge. Specifically, “explain,” “question,” and “figure out” are the cognitive activities unique to knowledge-for-teaching. The results demonstrate that when the participating teachers had doubts about the purpose behind the observed teaching practice, they tended to explain (19 times) or figure out (four times) the purpose rather than clarify their doubts *via* questioning (one time), a way to promote collaborative discussion. In addition, “assume,” a cognitive activity for the manipulation of knowledge, was characteristic of disciplinary knowledge. Table 5 shows that “assume” develops based on the surface processing *via* “describe.”

## Discussion

Now I answer the research questions.

### RQ1: In what manner did the participating teachers learn from the three demonstration lessons?

This question is addressed from two perspectives: cognitive activities in general and cognitive activities for specific teacher knowledge.

#### Cognitive activities in general

The teachers’ dominant use of “describe” as a cognitive activity and the rare involvement of “question” in their learning process suggest that the participating teachers perceived the modeled lessons as an object of study rather than a springboard for further teacher learning (O’Leary and Price, 2017). This is particularly evident in the interviewed teachers’ description of the teaching demonstrations as a means to provide a set of methods, activities, and techniques that would be directly applicable to their own classroom practice. As one teacher in the follow-up interview articulated, “the modelled lessons are a formula for teaching” and “establish a standard practice to follow” (I1T4^1^).

This perception of the modeled lessons may eventually constrain the participating teachers’ capacity to learn through observation. First, it could limit the development of further understanding of what was observed. Viewing themselves as “a doer” (Freeman, 2002, p. 5) who implemented “established patterns of thought and behaviour” (Deyrich and Stunnel, 2014, p. 90), the participating teachers learned from the modeled lessons, in many cases, only to the stage of making sense. This explains why “describe,” the cognitive activity for making sense, was employed in a much larger number than those for making meaning, including “explain,” “figure out,” and “question.”

Second, it probably caused the failure of the participating teachers to put what they learned into practice. The best possible representation of learning at the stage of making sense, as argued by Moon (1999), may be “simply reproducing the material” (p. 137). This is why the interviewed teachers suggested imitation as the best way to put the observed skills into practice. However, the process of making sense involves little connection to deeper or broader meanings, thereby leading the participating teachers to imitate the observed one without a clear understanding of his intended purpose in teaching. This can be seen in one teacher’s explanation of her unsuccessful teaching practice in the interview: “Later on, I made a close imitation of the first reading class, but it turned out to be a failure in the end. One reason for this might have been that I imitated the teacher, but I was not quite clear about his intention” (I3T1).

#### Cognitive activities for specific teacher knowledge

The tendency to employ the cognitive activities of “explain” and “figure out” rather than that of “question”—to erase any doubts about purpose in teaching—shows that the participating teachers viewed learning through observation more as an individual activity than as dialogic collaboration (Deyrich and Stunnel, 2014; O’Leary, 2014; Walsh, 2016; Barrell, 2017). This is supported by the results from the group interviews, where the teachers insisted on clarifying doubts by their own efforts, as illustrated by one teacher’s comment: “Learning through observation is a private and unnoticeable process which involves little or no discussion with the observed teacher” (I2T2).

The lack of initiative taken to interact with the observed teacher may well result in a restricted and mistaken interpretation of what was observed. This can be best illustrated by the following misinterpretation with regard to the purpose of classroom questioning in the modeled lessons where the teacher’s questions were primarily directed at providing students with opportunities to express themselves: “To ask good questions we should anticipate students’ answers at the time of lesson planning and meanwhile prepare several follow-up questions in case that the target words cannot be elicited from students” (V2T2U3).

With reference to the discussion above, it can be concluded that the participating teachers’ failure to question limited the use of observation to a means to learn practical skills rather than a medium on which to invite collaborative discussion to further teachers’ professional understanding. In other words, the absence of “question” as a cognitive activity during the learning process hindered the participating teachers from learning through observation in a collaborative and dialogic way.

### RQ1: In what manner did the participating teachers learn from the three demonstration lessons?

The present study identified four factors underlying the participating teachers’ failure to question.

The first factor was their perceived purpose of the modeled lessons. As discussed above, the participating teachers posited that the demonstrations aimed to offer “a formula for teaching” (I1T4), which “could be linked to specific learning outcomes” (Freeman and Johnson, 1998, p. 399), hence removing the need to further explore how teacher knowledge could be used for student learning. This clearly carries important implications for teacher education, suggesting that teacher educators need to help trainee teachers make use of the modeled lessons for professional development rather than try to understand the modeled lessons with a limited viewpoint or perspective.

The second factor was related to the participating teachers’ manner of making meaning. Their use of “explain” as a cognitive activity to make sense of what went on in the observed class suggests that the participating teachers were likely to make meaning *via* analogous experience (Dewey, 1910). This was later confirmed by the teachers in the interviews. When asked why they did not raise questions about the observed teacher’s purpose in teaching, the majority of the interviewed teachers stated that his intention was evident to them because they did the same thing in their own class. It indicates that teachers’ prior knowledge may prevent them from reflecting critically on what they observe. Accordingly, to help in-service EFL teachers adopt a critical stance to the pedagogical work they undertake requires teacher educators to have an awareness of teachers’ prior knowledge and of the difficulties they may have in discovering learning opportunities for students first and then provide mediation to facilitate critical reflection.

The third factor was associated with the participating teachers’ understanding of observer–observed relationship. A recurring concern in the interviews was the need to avoid critical feedback (e.g., questioning purpose in teaching) in order to maintain “harmonious” relationships between the observer and the observed. As one teacher expressed, “we are reluctant to question the observed teacher directly because we do not want to *offend* him” (I2T1). A similar finding was revealed in Hammersley-Fletcher and Orsmond’s (2005) study, where the observers reported feelings of anxiety about providing “critical feedback” to the demonstration teacher. It shows that a cooperative and constructive observer–observed relationship seems essential to learning through observation. To facilitate such a relationship, teacher educators need to create a climate of learning and sharing for the post-observation discussion sessions.

The fourth factor was linked to the participating teachers’ perception of professional learning. A common consensus among the interviewed teachers was that when doubts about purpose in teaching arose from their observation, they were inclined to remove these doubts *via* the cognitive activity of “explain” or “figure out.” The former involves “interpreting what was observed through prior understandings” (I1T3), while the latter entails “observing the teacher attentively, thinking about the relations between each step, and then eliciting a reasonable explanation for his teaching based on the observation” (I2T1). The application of “explain” and “figure out” reveals that the participating teachers conceived professional learning as “an individual act or the sole responsibility of the teacher” (O’Leary, 2014, p. 116). It suggests that learning through observation, like student learning, needs learner training. In this regard, it might be important for teacher educators to give a training session on learning strategies prior to lesson observation.

## Conclusion

The manner in which EFL teachers learn through lesson observation has been sparsely addressed in the literature. With the help of non-mediated verbal reporting and semi-structured group interviews, the present study examined the nature of 32 Chinese senior high school EFL teachers’ learning through observation. Exploring how the participating teachers directed their attention, processed what they observed, and made sense of the teaching practice in the modeled lessons, the findings of this study indicate that the cognitive activity of “question,” which is believed to be instrumental in generating collaborative discussion, was often absent from the participating teachers’ learning processes and such an absence reduced their learning to the study of practical skills, which could be conducted in isolation. Contributing factors to the participating teachers not adopting a shared and dialogic approach to learning through observation included their perceived purposes of the modeled lessons, their manner of making meaning, their understanding of observer–observed relationship, and their perception of professional learning.

Investigating the participating teachers’ learning processes not only sheds light on their way of learning, but also sensitizes teacher educators to the factors that may influence in-service EFL teachers’ learning through observation and the difficulties that they may encounter during the learning process. Recognizing these influential factors and teachers’ potential difficulties is obviously an important starting point for quality mentorship. While previous research has explored the role of teacher educators in shaping EFL teachers’ reflection after observation (e.g., Yuan and Lee, 2014; Engin, 2015), scant attention has been paid to a teacher educator’s mentorship in relation to the factors that may have an effect on teacher learning through observation. Future studies may find it beneficial to examine how teacher educators foster teacher learning with regard to these influencing factors.

Given its significant role in teacher education, learning through observation is a topic that necessitates further investigation. This study concentrated on learning through observation in the context of an in-service teacher training program. Future research could further explore the topic in varying contexts, such as an initial teacher training course or a collaborative professional development initiative.

With respect to limitations, this study was limited in scope to only 32 in-service EFL teachers in the same district. Hence, while insights may be drawn, there may be difficulties in generalizing the findings beyond the immediate context. Further studies should encompass a broader range of teachers and contexts to determine with more certainty what distinguishes how EFL teachers in China learn through lesson observation.

## Data availability statement

The original contributions presented in the study are included in the article/supplementary material, further inquiries can be directed to the corresponding author.

## Ethics statement

Ethical review and approval was not required for the study on adult human participants in accordance with the local legislation and institutional requirements. Written informed consent for participation was not required for this study in accordance with the national legislation and the institutional requirements.

## Author contributions

The author confirms being the sole contributor of this work and has approved it for publication.

## Funding

This work was supported by Science Foundation of Zhejiang Sci-Tech University (22112279-Y), and General Research Project of the Department of Education of Zhejiang Province, China (Y201738248).

## Conflict of interest

The author declares that the research was conducted in the absence of any commercial or financial relationships that could be construed as a potential conflict of interest.

## Publisher’s note

All claims expressed in this article are solely those of the authors and do not necessarily represent those of their affiliated organizations, or those of the publisher, the editors and the reviewers. Any product that may be evaluated in this article, or claim that may be made by its manufacturer, is not guaranteed or endorsed by the publisher.

## Footnotes

^1^The abbreviations "I" for a group interview and "T" for an identified teacher in a group interview are used to identify quotes from the interview data. For example, the abbreviation "I1T4" refers to a quote from a teacher who was the fourth one to take the floor in the first interview.

## Appendix

Appendix: The interview outline

1. What do you often attend to when observing demonstration lessons?
2. Why do you focus on those aspects?
3. Have you ever questioned the observed teachers about their purpose in teaching? If not, please explain your reason.
4. Have you applied what you learned from the three demonstration lessons to your practices? If yes, please illustrate your application with detailed examples.
5. What is the effect of the three demonstration lessons on your teaching practices?
